# Towards autoregulation-oriented management after traumatic brain injury: increasing the reliability and stability of the CPPopt algorithm

**DOI:** 10.1007/s10877-023-01009-1

**Published:** 2023-04-29

**Authors:** Erta Beqiri, Ari Ercole, Marcel J. H. Aries, Michal M. Placek, Jeanette Tas, Marek Czosnyka, Nino Stocchetti, Peter Smielewski, CENTER-TBI High Resolution (HR ICU) Sub-Study Participants and Investigators, Audny Anke, Ronny Beer, Bo-Michael Bellander, Erta Beqiri, Andras Buki, Manuel Cabeleira, Marco Carbonara, Arturo Chieregato, Giuseppe Citerio, Hans Clusmann, Endre Czeiter, Marek Czosnyka, Bart Depreitere, Ari Ercole, Shirin Frisvold, Raimund Helbok, Stefan Jankowski, Daniel Kondziella, Lars-Owe Koskinen, Ana Kowark, David K. Menon, Geert Meyfroidt, Kirsten Moeller, David Nelson, Anna Piippo-Karjalainen, Andreea Radoi, Arminas Ragauskas, Rahul Raj, Jonathan Rhodes, Saulius Rocka, Rolf Rossaint, Juan Sahuquillo, Oliver Sakowitz, Peter Smielewski, Nino Stocchetti, Nina Sundström, Riikka Takala, Tomas Tamosuitis, Olli Tenovuo, Andreas Unterberg, Peter Vajkoczy, Alessia Vargiolu, Rimantas Vilcinis, Stefan Wolf, Alexander Younsi, Frederick A. Zeiler

**Affiliations:** 1 Brain Physics Laboratory, Division of Neurosurgery, Department of Clinical Neurosciences, University of Cambridge, Cambridge, UK; 2 Division of Anaesthesia, University of Cambridge, Cambridge, UK; 3 Department of Intensive Care Medicine, Maastricht University Medical Center+, Maastricht, The Netherlands; 4 School of Mental Health and Neurosciences, Maastricht University, Maastricht, The Netherlands; 5 Institute of Electronic Systems, Warsaw University of Technology, Warsaw, Poland; 6 Department of Anaesthesia and Critical Care, Neuroscience Intensive Care Unit, Fondazione IRCCS Ca’ Granda-Ospedale Maggiore Policlinico, Milan, Italy; 7 Department of Pathophysiology and Transplants, University of Milan, Milan, Italy

**Keywords:** CPPopt, Cerebral autoregulation, Traumatic brain injury, Reliability, Stability, Multiwindow weighted approach

## Abstract

**Purpose:**

CPPopt denotes a Cerebral Perfusion Pressure (CPP) value at which the Pressure-Reactivity index, reflecting the global state of Cerebral Autoregulation, is best preserved. CPPopt has been investigated as a potential dynamically individualised CPP target in traumatic brain injury patients admitted in intensive care unit. The prospective bedside use of the concept requires ensured safety and reliability of the CPP recommended targets based on the automatically-generated CPPopt. We aimed to: Increase stability and reliability of the CPPopt automated algorithm by fine-tuning; perform outcome validation of the adjusted algorithm in a multi-centre TBI cohort.

**Methods:**

ICM + software was used to derive CPPopt and fine-tune the algorithm. Parameters for improvement of the algorithm were selected based on qualitative and quantitative assessment of stability and reliability metrics. Patients enrolled in the Collaborative European Neuro Trauma Effectiveness Research in TBI (CENTER-TBI) high-resolution cohort were included for retrospective validation. Yield and stability of the new algorithm were compared to the previous algorithm using Mann–U test. Area under the curves for mortality prediction at 6 months were compared with the DeLong Test.

**Results:**

CPPopt showed higher stability (*p* < 0.0001), but lower yield compared to the previous algorithm [80.5% (70—87.5) vs 85% (75.7—91.2), p < 0.001]. Deviation of CPPopt could predict mortality with an AUC of [AUC = 0.69 (95% CI 0.59–0.78), p < 0.001] and was comparable with the previous algorithm.

**Conclusion:**

The CPPopt calculation algorithm was fine-tuned and adapted for prospective use with acceptable lower yield, improved stability and maintained prognostic power.

## Introduction

Cerebral autoregulation (CA) maintains an adequate and relatively constant cerebral blood flow (CBF) despite changes in cerebral perfusion pressure (CPP) [1, 2]. The autoregulatory response is frequently impaired in the acute phase after severe traumatic brain injury (TBI) [3, 4]. The clinical importance of a preserved CA in TBI is related not only to the ability of ensuring adequate blood flow to an injured brain in the face of inevitable variations of CPP, but also because of the possibility open to clinicians of interacting with the vasodilatory cascade involved in intracranial hypertension episodes in severe TBI. Therefore, targeting CPP to values that would optimise autoregulation emerges as a promising strategy in the critical care management of TBI patients.

Optimal CPP or CPPopt denotes a Cerebral Perfusion Pressure (CPP) value at which the Pressure-Reactivity index (PRx) [3, 5], reflecting the global state of CA, is best preserved. CPPopt is conceptually defined as the CPP value corresponding to the optimum of the U-shape curve that describes the relationship between PRx and CPP [4] over time. When studied in a large cohort of TBI patients, CPPopt showed a significant relationship with outcome: worse outcome was related to deviation of CPP from CPPopt. In particular, mortality increased when CPP was lower than CPPopt and disability increased when CPP was higher than CPPopt [6].

Achieving a continuous automated assessment of individualised CPPopt over hours rather than days was a key step towards clinical use. The first automated algorithm published by Aries et al. [6] was based on fitting a second order polynomial to PRx and CPP data obtained from a four hour moving window. The minimum of this U-shaped relationship determines CPPopt. The calculations were updated every minute to track CPPopt over time. This algorithm had a yield of on average 55% of the time. Efforts to increase the yield of the algorithm and therefore its potential clinical applicability lead to the multi-window approach [7]—with a CPPopt yield over 90%.

Availability alone is not everything. There are no physiological measurements that are entirely reliable or statistically stationary. As a result, any derived parameter such as CPPopt is also subject to perturbations and noise. Therefore, any algorithm must additionally evaluate a level of confidence in the result so that when this falls below some threshold, the calculations can be rejected. Conceptually this is analogous, for example, to a clinician ‘eyeballing’ a waveform and making a judgement on physiological data quality. It follows that, for an automated system, there are two possible reasons why a prototype algorithmic refinement may appear to increase the yield of CPPopt measurements. Firstly, this may be because the algorithm is better able in discriminating signal from noise. This is desirable and may be achieved by sophisticated, but complex and potentially highly parameterizable, filtering techniques (such as the multi-window). Secondly, but highly undesirable, it may result from a relaxation of the threshold below which the algorithm rejects data about which it is uncertain.

No measurement is perfect and conceptual trade-offs between true and false positive results on the basis of some threshold are common place in diagnostic medicine- it is not a dichotomous decision. Analogously, whether to favour CPPopt availability or discriminate against uncertainty at the expense of yield will depend to a large extent on the application- there is no single solution. For a clinical feasibility and safety trial [8] for example, it is important to be appropriately conservative given experience to date and then applying surveillance to the prospective data to ensure safety. Whether a better solution can be devised by further algorithmic optimization for a future putative clinical tool requires more careful evaluation and is an incremental process.

Here we present the efforts of the fine-tuning process that aimed to increase stability and reliability of the CPPopt automated algorithm in order to adjust it for prospective bedside use. The objective of the fine-tuning process was to increase stability and reliability without large reductions in availability of the CPPopt values generated by the algorithm.

Single centre retrospective outcome validation of this adjusted algorithm was published by our group previously [9, 10], and the algorithm was used in the COGITATE study (registered as NCT02982122 in ClinicalTrials.gov) [8]. Here we show results of a large multicentre retrospective validation.

The main objective of the statistical validation was to confirm in a multicentre cohort that the modifications implemented during the fine-tuning process had increased stability of the automated algorithm when compared to the previous one and to explore the effect on yield. The secondary objective was to assess whether the outcome predictive power was still maintained.

## Methods

### Algorithm fine-tuning process

#### Multi-window weighted approach as implemented in the previous algorithm

PRx is calculated as a moving correlation coefficient between 10 s averages of intracranial pressure (ICP) and arterial blood pressure (ABP) waveforms in a window of 5 min [5]. The trendline of Optimal cerebral perfusion pressure (CPPopt) is calculated using a multi-window approach inspired by Depreitere et al. [11], subsequently implemented in ICM + [12] (https://icmplus.neurosurg.cam.ac.uk) and investigated in a retrospective TBI data set by Liu et al. [7]. At each time point, 36 PRx-cerebral perfusion pressure (CPP) plots are generated from past data windows of increasing duration ranging from 2 to 8 h, using incremental steps of 10 min. Prior to that, CPP time series are pre-processed with a 5 min duration mean filter and PRx data are Fisher transformed to ensure normal distribution of PRx values and remove the bias due to the < -1; + 1 > constraints [13]. Subsequently, all the data points are divided into groups corresponding to CPP bins of 5 mmHg length, within 40–120 mmHg range of CPP values. Mean PRx and CPP values from each bin are used to fit a second order polynomial describing the theoretical U-shape, with its nadir determining CPPopt. This process is repeated for each progressively longer data window. Individual results undergo certain quality control criteria and the accepted values are combined using a weighted average operation. The calculations are repeated every minute and the resulting time series is finally subjected to an exponentially weighted average (EWA) filter of 2 h of duration forming the CPPopt time trend.

#### Main problems with the previous algorithm

The algorithm published by Liu et al. [7] (named here ‘CPPopt_MA’) was tested on retrospective datasets, in which artefacts were removed manually and in which CPP was generally not managed according to CPPopt. Moreover, CPPopt ‘false positive’ values can be generated from non-physiological variations of ICP and ABP [14]. As a result, the pilot prospective run of the CPPopt_MA algorithm at the bedside highlighted instability of the time trend and occurrence of non-physiological CPPopt values. These are unacceptable when the CPPopt values are to be used as CPP target recommendation in individual patients prospectively.

#### Parameters and heuristics

In the multi-window weighted based algorithm implemented in ICM + (https://icmplus.neurosurg.cam.ac.uk) [7] there are mainly two sets of parameters that could be fine-tuned. The first is represented by the PRx-CPP curve fitting criteria. Table 1 shows the list of parameters that were available and their original setting.

**Table 1:** List of the curve fitting parameters available in ICM + and their settings before and after the fine-tuning process was performed

Parameter	Description	CPPopt_MA	CPPopt
Arguments	X and y axis of the plots. PRx data are Fisher transformed (y axis – PRx (ft)). CPP time series represent the x axis. In the current algorithm CPP time series are pre-processed with a 5 min duration Median filter (CPPmedian_5min)	CPP mean; PRx (ft)	CPPmedian_5min; PRx (ft)
Missing data limit	Percentage of missing data values in the calculation window below which the calculation will not be performed	50%	50%
Number of bins	A CPP bin is the range of values that are stored as one unit. When the minimum bin is 40 mmHg and the maximum bin is 120 mmHg and the number of bins is set at 16, the CPP bin width is 5 mmHg	16 subsets	16 subsets
Minimum bin value	CPP bins below this minimum value are not used for curve fitting	40 mmHg	40 mmHg
Maximum bin value	CPP bins above this maximum value are not used for curve fitting	120 mmHg	120 mmHg
Minimum bin data count [%]	Relative minimum number of data points included in the bin for it to be available for the curve fitting	1–2%	3%
Minimum included data [%]	Relative minimum number of data points that has to be covered by the fitted curve	50%	50%
Minimum Y span	Minimum span of PRx covered by the fitted curve. In other words, too flat curves (PRx span < 0.2) are rejected	0.2	0.2
Enforce Y-region – Min	This option sets the value for the LOWER border of the PRx range that the fitted curve must overlap	− 0.3	− 0.3
Enforce Y-region – Max	This option sets value for the UPPER border of the PRx-range that the fitted curve must overlap	0.6	0.6
R^2^_full_	Coefficient of determination of the fitted curve, calculated using all the bins, also including the ones rejected from the curve fitting process	(–)	0.2

A brief description and the original value set for the curve fitting parameters in the previous algorithm (CPPopt_MA) are shown and compared to the current settings (CPPopt). CPP, cerebral perfusion pressure. PRx, pressure reactivity index

The second group of parameters is represented by the settings of the weighting process. In the original CPPopt_MA algorithm the weight was calculated as

$$\mathrm{Weight}= \frac{1}{{\mathrm{e}}^{\mathrm{window lenght}}}\mathrm{ x }\frac{1}{{\mathrm{e}}^{\mathrm{full fit error}}}\mathrm{ x }{\mathrm{W}}_{\mathrm{Non Parabolic window}}$$

The arguments of the function were the mean value of CPP calculated over a 5-min moving window and transformed PRx.

#### Fine-tuning

Different sets of curve-fit and weighting heuristics were tested on two training data sets: 1) prospectively collected ABP and ICP waveforms of 5 TBI patients; 2) 25 realisations of data generated from randomised, surrogate, signals from the ABP and ICP waveforms of the same patients (generated noise). The latter was achieved by using the original data signals, Fourier transform the signals and then randomising the phase components, before applying an inverse transform. In this way, the generated signals retained the same dynamic range as the original signals, but the relationship between them was completely destroyed making them suitable for direct comparison with the original set [15].

ICP and ABP waveforms (and the corresponding generated noise) were processed with ICM + software. Different combinations of curve-fit and weighting heuristics were evaluated qualitatively and quantitatively by two researchers (EB and PS) in terms of their performance in generating plausible CPPopt values (> 50 mmHg and < 100 mmHg) and generating stable trendlines of CPPopt with less jumps, where these were defined as an abrupt difference of > 10 mmHg in less than 5 min. Reliability of the generated output was assessed by looking at the correspondence between the automatically generated value and the optimum of the manually plotted PRx/CPP curves. Finally, the reliability was also assessed via the ability of the algorithms in rejecting CPPopt calculations resulting from generated noise. Based on these evaluations, the best performing parameters were selected and approved by a larger group of researchers (EB, AE, MA and PS).

#### Fine-tuning output

The fine-tuning process led to the CPPopt algorithm that was used in the COGiTATE prospective trial [8]. In the protocol paper [16] of the trial we described the main features of this algorithm. They are available in www.cppopt.org and are reported here for completeness.

**Fig. 1:**
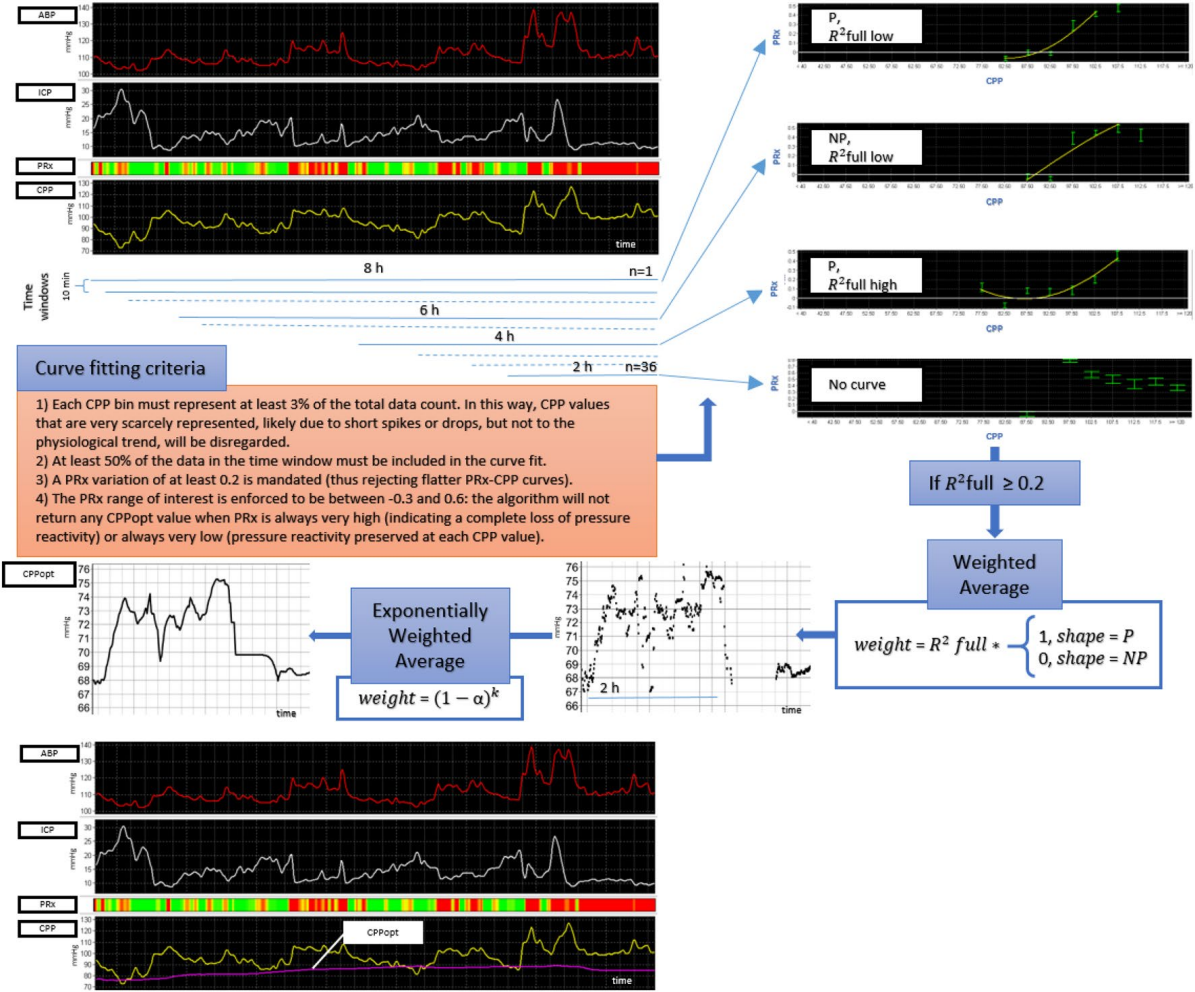
CPPopt updated algorithm. A schematic representation of the revised multi-window weighted approach is depicted. The charts at the left top show an 8 h screenshot of ABP and ICP min by min processed trends. CPP is calculated as ABP – ICP. PRx is displayed as a risk bar chart with green colour representing negative values and red colour representing positive values. The data of this time period are divided into 36 time windows. For each time window, PRx/CPP error bar charts are derived according to the curve fitting criteria listed in the orange box. At the top right four examples of PRx/CPP error bar charts are shown and here described from top to bottom. In the first chart the second order polynomial fit shows a parabolic curve but with low R^2^_full_. This curve will have a low weight. The second curve is non parabolic and therefore will be rejected. The third curve is parabolic with a high R^2^_full_, therefore the optimum of this curve will have a high weight. In the fourth plot no curve could be fitted. The diagram shows that the next check point would be the value of R^2^_full_ with a threshold of 0.2. The optimum of the curves will then undergo the weighted average process, where only parabolic curve will be accepted, R^2^_full_ being the weighting factor. Data points of two hours undergo an exponentially weighted average. In the left bottom box, the CPPopt time trend is shown in the bottom chart. Further details of the algorithm are described in the main text of this manuscript (results- section I). ABP, arterial blood pressure; ICP, intracranial pressure; PRx, pressure reactivity index; CPP, cerebral perfusion pressure; R^2^_full_, determination coefficient calculated for all the bins; P, parabolic intended as U-shaped; NP, non-parabolic intended as non-U-shaped

In the new algorithm (Fig. 1 and Table 1), the arguments of the function are minute-by-minute time series of median value of CPP (instead of the mean value) calculated over a 5-min moving window and PRx calculated over the same time window.

The curve fitting process for each time window follows the algorithm described by Aries et al. [17] with additional criteria as follows:

1. Each CPP bin must represent at least 3% of the total data count. In this way, CPP values that are very scarcely represented, likely due to short spikes or drops, but not to the physiological trend, will be disregarded. In the previous algorithm the parameter was set at 2%.
2. At least 50% of the data in the time window must be included in the curve fit.
3. A PRx variation of at least 0.2 is mandated (thus rejecting flatter PRx-CPP curves).
4. The PRx range of interest is enforced to be between − 0.3 and 0.6: the algorithm will not return any CPPopt value when PRx is always very high (indicating a complete loss of pressure reactivity) or always very low (pressure reactivity preserved at each CPP value).
5. The coefficient of determination of the fitted curve R^2^_full_ (calculated also for the bins excluded from the curve fitting process) must be at least 0.2. This feature was not available in the previous algorithm.

The weights for combining the CPPopt calculations are given by the following formula:

$$\mathrm{Weight}={\mathrm{R}}_{\mathrm{full}}^{2} *\left\{\begin{array}{c}1, shape=P\\ 0, shape=NP\end{array}\right.$$

where P and NP stand for parabolic and non-parabolic, respectively.

The exponentially weighted average (EWA) weight is calculated as (1-α) ^k^ where k is the distance, in number of samples, from the current sample and α is set at 0.1. In this way, more recent CPPopt values contribute more to the final calculation. The missing data limit of the calculation is set at 50%, therefore at least 4 h of continuously acquired data are necessary to generate the first CPPopt value.

### Multicentre-retrospective validation

The goal of this investigation was to assess the performance of the new algorithm when compared to the previous algorithm in a small but multi-centre cohort of patients admitted in the intensive care unit (ICU). For clarity, the new algorithm will be referred here as ‘CPPopt’, For this multi-centre validation investigation we used the Collaborative European Neuro Trauma Effectiveness Research in TBI (CENTER-TBI) study high-resolution (HR) ICU sub-study cohort [18].

#### Material

We considered 277 patients enrolled in the High-Resolution cohort of the Collaborative European Neuro Trauma Effectiveness Research in TBI (CENTER-TBI) high-resolution ICU sub-study [19] over 21 recruiting centres from 2014 to 2017. All patients were admitted to ICU for their TBI during the course of the study. High resolution digital signals were recorded from their ICU monitors during the course of their ICU stay.

The CENTER-TBI study (EC grant 602,150) has been conducted in accordance with all relevant laws of the EU if directly applicable or of direct effect and all relevant laws of the country where the Recruiting sites were located, including but not limited to, the relevant privacy and data protection laws and regulations (the “Privacy Law”), the relevant laws and regulations on the use of human materials, and all relevant guidance relating to clinical studies from time to time in force including, but not limited to, the ICH Harmonised Tripartite Guideline for Good Clinical Practice (CPMP/ICH/135/95) (“ICH GCP”) and the World Medical Association Declaration of Helsinki entitled “Ethical Principles for Medical Research Involving Human Subjects”. Informed Consent by the patients and/or the legal representative/next of kin was obtained, accordingly to the local legislations, for all patients recruited in the Core Dataset of CENTER-TBI and documented in the e-CRF.

Ethical approval was obtained for each recruiting site. The list of sites, Ethical Committees, approval numbers and approval dates can be found on the website: https://www.center-tbi.eu/project/ethical-approval

Data for the CENTER-TBI study has been collected through the Quesgen e-CRF (Quesgen Systems Inc, USA), hosted on the INCF platform and extracted via the INCF Neurobot tool (INCF, Sweden). For patient monitoring and data collection in the High-Resolution repository, the ICM + platform (Cambridge Enterprise Ltd, Cambridge, UK) and/or Moberg Neuromonitoring system (Moberg Research Inc., USA) were used.

Detailed data collection and pre-processing methods (artefact cleaning and down-sampling to 10 s averaged time series) applied to high resolution data of the cohort considered for our study have been described in preceding works [20, 21]. Arterial Blood Pressure (ABP) and intracranial pressure (ICP) 10 s averaged series were retrieved for this analysis.

The following information was accessed using Opal software [22] and Neurobot (data release v 3.0) on the 15^th^ March 2021: clinical outcome as assessed by the Glasgow Outcome Score Extended (GOSE) at 6 months; external ventricular drain (EVD) insertion time, decompressive craniectomy (DC) time, death time. Further data processing and statistical analysis were performed with ICM + software and R statistical language version 4.0 [23].

We considered different time periods depending of the sub analysis. For the stability and yield analysis we considered the first seven days from the start of the recording. Stability and yield are related to calculation’s properties and in particular to the data buffer considered. For the outcome analysis we considered the first seven days from the time of injury as we investigated a clinical relationship.

In both sub analysis we excluded patients with ICP monitored via EVD (n = 37), due to their poor ICP data quality, or that underwent a DC during the monitoring periods considered (n = 37 for the stability-yield analysis; n = 39 for the outcome analysis) as the validity of PRx in those cases is still not fully established. Eighteen patients did not have GOSE outcome assessment at 6 months and their survival status is not known. Hence, they were not considered in the outcome analysis.

#### Measurements

ABP and ICP 10 s averaged series were processed with ICM + software to derive CPPopt (and CPPopt_MA) time trends.

Stability of the time trends was assessed with the stability index, which was calculated as the standard deviation of the difference of two consecutive values of CPPopt (or CPPopt_MA). The stability index gives a measure of short-term variability. A lower value of the index means a higher stability.

Yield was calculated as the percentage of total CPP recorded time with CPPopt (or CPPopt_MA) values available (%). DeltaCPPopt (and DeltaCPPopt_MA) was calculated as the average deviation of CPP from CPPopt (or CPPopt_MA).

#### Statistical analysis

Normality of continuous variables was assessed with histograms, quantile–quantile plots and the Shapiro-Wilks test.

Mann-U test was used for comparing between algorithms stability index and yield. Kruskall-Wallis test was used for comparing between algorithms stability index across multiple days and for comparing CPPopt relationship with GOSE categories. Outcome groups were identified using GOSE score. Mortality was defined by GOSE = 1. Unfavourable outcome was defined by GOSE between 1 and 4. The relationship with dichotomised outcome (alive vs dead and favourable vs unfavourable) was assessed with Mann-U test and logistic regression models. AUC (CI 95%) were calculated and compared with the DeLong Test.

Whole 7 days periods and daily periods were considered separately.

## Results

### Algorithm Fine-tuning process

**Fig. 2:**
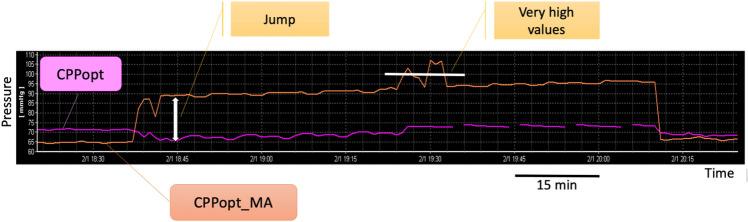
Example of discontinuities and very high values in CPPopt trend. CPPopt trend calculated with the previous algorithm (CPPopt_MA, in orange) shows a sudden jump from 65 to 95 mmHg in less than 5 min. The high values are maintained for over two hours. Moreover, the trend spikes further and reaches very high values, above 100 mmHg (white horizontal bar). CPPopt calculated with new algorithm (in pink) from the same data, but with more restrictive criteria, is instead very stable and doesn’t display the erratic jumps behaviour. CPPopt, optimal cerebral perfusion pressure

**Fig. 3:**
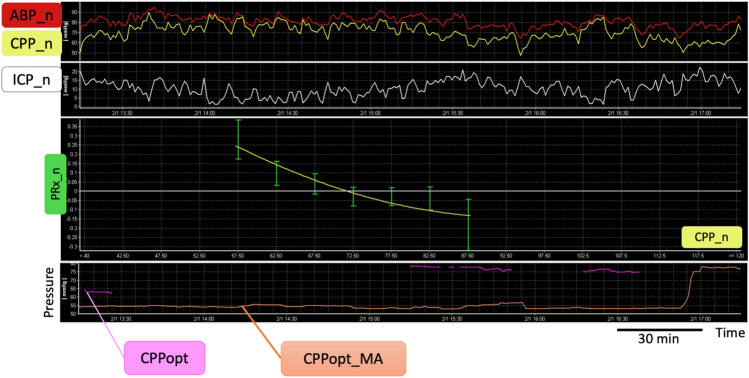
Comparison between the new and previous algorithm in deriving CPPopt from generated noise. The top charts show 4 h of arterial blood pressure, intracranial pressure and cerebral perfusion pressure min by min trends generated form surrogate signals (generated noise). The error bar chart in the middle shows a second order polynomial curve fit on these surrogate data that would allow to identify an optimum and therefore a value for ‘CPPopt’ that would represent a false-positive value. In the bottom chart the CPPopt time trend generated with the multi-window weighted approach is shown, comparing the new (CPPopt in pink) and previous algorithm (CPPopt_MA in orange). The previous algorithm would give false positive CPPopt values for the whole period. The new algorithm reduces the amount of false positive data. Ideally, no CPPopt should be calculated from generated noise. ABP_n, arterial blood pressure noise; ICP_n, intracranial pressure noise; CPP_n, cerebral perfusion pressure noise; PRx_n, pressure reactivity noise; CPPopt, optimal cerebral perfusion pressure

Altogether, the features selected during the fine-tuning process made it possible to decrease the number of abrupt jumps of the time trends. Figure 2 shows an example of comparison between the previous (CPPopt_MA) and the new (CPPopt) algorithm on this aspect. Furthermore, these features ensured better reliability of the values in reflecting the status of cerebral autoregulation. Figure 3 shows an example illustrating that the new algorithm reduces the total number of values calculated from generated noise. The figure also shows that, as a consequence, CPPopt yield (availability of CPPopt values in time) was reduced. In fact, given the imposed restrictions, there would be less CPPopt values calculated. The new algorithm was used in the COGiTATE prospective trial [8].

### Retrospective multicentre validation

#### Stability and yield analysis

In 3 cases we could not calculate any value of CPPopt within the first 7 days of recordings. Hence the total number of recordings included in the stability and yield analysis was 198.

**Fig. 4:**
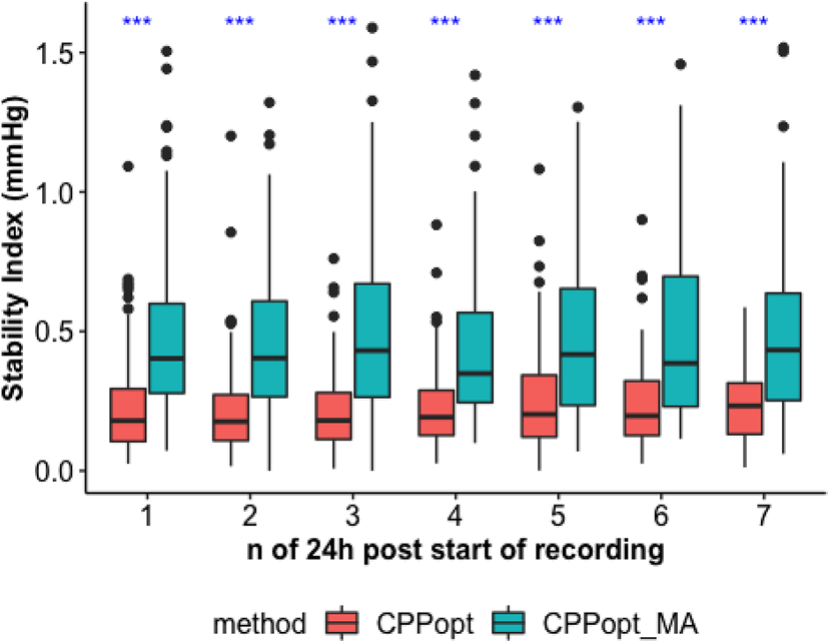
Stability index for CPPopt and CPPopt_MA. Stability index boxplots are assessed for the two calculation methods (CPPopt and CPPopt_MA) and compared for each 24 h epoch from the beginning of the recording. Blu asterisks stand for significant difference between the two algorithms (Kruskal–Wallis test, *p* < 0.001). CPPopt: algorithm resulted after the fine-tuning process. CPPopt_MA: previously available algorithm

CPPopt stability over the whole 7 days recording period (Table 2) was significantly higher (lower stability index) when compared to CPPopt_MA (Mann-U test, *p* < 0.001). The difference in stability between the two methods appeared to be significant (Kruskal–Wallis test,* p* < 0.001) across the whole recording period (Fig. 4 and Table 2).

**Table 2:** Stability analysis descriptive results

Variable		N	Median	IQR
Whole period				
	CPPopt	198	0.21	(0.16–0.27)
	CPPopt_MA	198	0.45	(0.34–0.62)
Day 1				
	CPPopt	192	0.18	(0.11–0.29)
	CPPopt_MA	192	0.4	(0.28–0.6)
Day 2				
	CPPopt	188	0.18	(0.11–0.27)
	CPPopt_MA	188	0.4	(0.27–0.61)
Day 3				
	CPPopt	169	0.18	(0.11–0.28)
	CPPopt_MA	169	0.43	(0.26–0.67)
Day 4				
	CPPopt	143	0.19	(0.13–0.29)
	CPPopt_MA	143	0.35	(0.24–0.57)
Day 5				
	CPPopt	119	0.2	(0.12–0.34)
	CPPopt_MA	119	0.42	(0.23–0.65)
Day 6				
	CPPopt	81	0.2	(0.13–0.32)
	CPPopt_MA	81	0.39	(0.23–0.7)
Day 7				
	CPPopt	58	0.23	(0.13–0.31)
	CPPopt_MA	58	0.43	(0.25–0.64)

Stability index of the trends generated by the two methods are reported with median values and IQR. Whole period refers to the first 7 days from the beginning of the recording. A lower value of the index means higher stability. See Fig. 4 for statistical comparisons. CPPopt: algorithm resulted after the fine-tuning process. CPPopt_MA: previously available algorithm

**Fig. 5:**
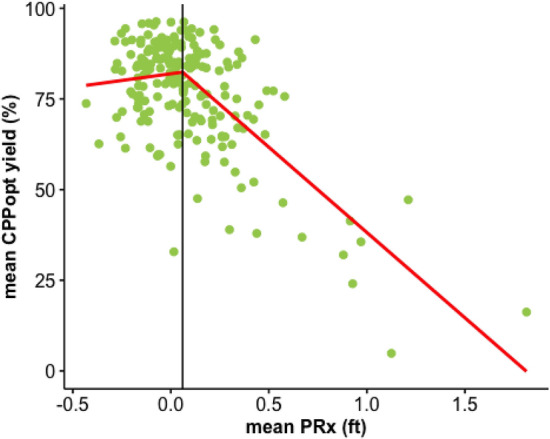
Relationship between patients’ average CPPopt yield and PRx. The scatterplot shows the relationship between average CPPopt yield and average PRx. Fisher transformation was applied to PRx (PRx (ft) = PRx fisher transformed) before calculating average value for each patient. In the plot, each point represents one patient. The breakpoint identified with piecewise linear model was at PRx (ft) = 0.06. CPPopt: optimal cerebral perfusion pressure assessed with the algorithm resulted after our fine-tuning process

On average CPPopt was available after 5 h (4–8) starting from the beginning of the recording. Median (IQR) CPPopt yield (80.5% (70—87.5)) was significantly lower than CPPopt_MA yield (85% (75.7—91.2), *p* < 0.001) for the first 7 days of neuromonitoring. The relationship between average CPPopt yield and ICP, CPP and PRx was investigated with scatterplots and piecewise linear regression if appropriate. Levels of ICP and CPP seemed not to influence CPPopt yield, but high levels of PRx were related to low CPPopt yield (Fig. 5).

#### Outcome analysis—whole 7 days period form the time of injury

In 18 cases outcome assessment was not available. One patient didn’t have ICP monitoring within the first 7 days from injury, but only afterwards. In 2 patients CPPopt (and CPPopt_MA) could not be calculated. Therefore, the total number of patients included in the outcome analysis was 182.

**Fig. 6:**
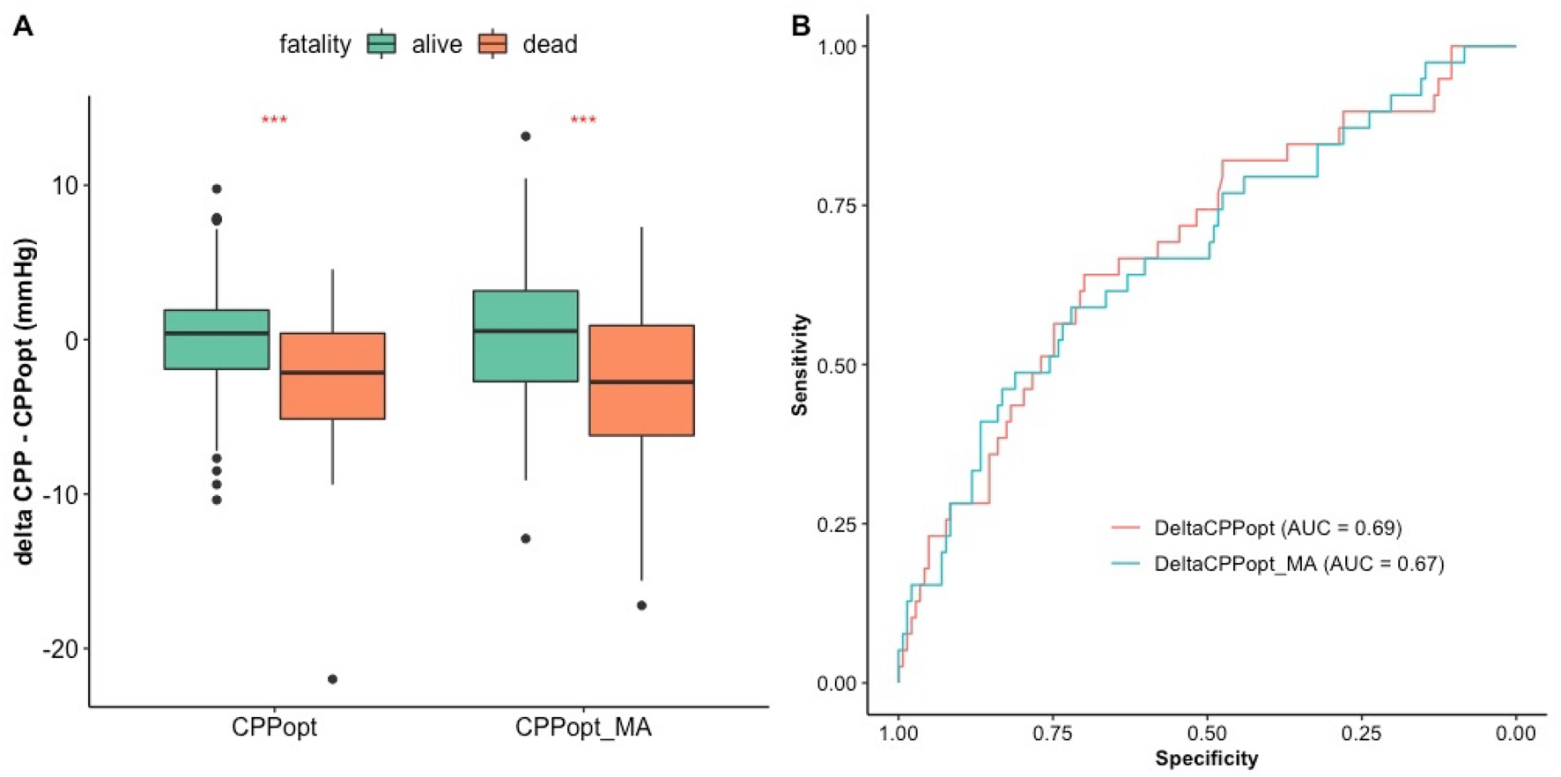
Average deviation of CPP from CPPopt compared to mortality groups for each calculation method and their univariate logistic regression ROC curves for mortality prediction. **A** The figure shows boxplots of mean deviation of CPP from CPPopt for each calculation method (CPPopt and CPPopt_MA) and for two mortality groups (alive and dead, as assessed by GOSE at 6 months). **B** Univariate logistic regression receiver operator characteristic curves for mortality prediction for average deviation of CPP from CPPopt and from CPPopt_MA. The first 7 days from the day of injury are considered. CPP: cerebral perfusion pressure. CPPopt: optimal cerebral perfusion pressure assessed with the algorithm resulted after our fine-tuning process. CPPopt_MA: optimal cerebral perfusion pressure assessed with the previously available algorithm. DeltaCPPopt: average deviation of cerebral perfusion pressure from optimal cerebral perfusion pressure assessed with the algorithm resulted after our fine-tuning process. DeltaCPPopt_MA: average deviation of cerebral perfusion pressure from optimal cerebral perfusion pressure assessed with the previously available algorithm

Average deviation of CPP from both CPPopt and CPPopt_MA was significantly different between mortality groups (Kruskall-Wallis test, *p* < 0.001), as shown in Fig. 6 (panel A) and Tables 2, 3.

**Table 3:** Outcome analysis descriptive results

Mortality group	Method	N	Median	IQR
Alive	CPPopt	143	0.40	(− 1.9 to 1.91)
Alive	CPPopt_MA	143	0.55	(− 2.71 to 3.16)
Dead	CPPopt	39	− 2.14	(− 5.14 to 0.41)
Dead	CPPopt_MA	39	− 2.75	(− 6.21 to 0.91)

Median and IQR values of delta CPP − CPPopt (denoted with method = CPPopt) and delta CPP − CPPopt_MA (denoted with method = CPPopt_MA) are presented for two mortality groups (alive and dead) as assessed by GOSE at 6 months. CPPopt: algorithm resulted after the fine-tuning process. CPPopt_MA: previously available algorithm

Mann-U test (*p* value < 0.001) and univariate logistic regression (AUC = 0.69 (95% CI 0.59–0.78), *p* < 0.001) showed that DeltaCPPopt could distinguish mortality groups. DeLong test (*p* = 0.67) confirmed that DeltaCPPopt performed no worse than DeltaCPPopt_MA (AUC = 0.67 (0.58–0.78), *p* < 0.001). Panel B in Fig. 6 shows receiver operator characteristic curves for mortality prediction for both methods.

Mann-U test didn’t show significant difference in delta CPPopt (*p* = 0.06) nor CPPopt_MA (*p* = 0.05) when comparing favourable and unfavourable outcome. Univariate logistic regression showed that the ability of DeltaCPPopt (AUC = 0.58 (0.50—0.66), *p* = 0.03) and DeltaCPPopt_MA (AUC = 0.58 (0.50—0.67), *p* = 0.03) in predicting favourable and unfavourable outcome could just reach statistical significance, without significant difference between the two (DeLong Test, *p* = 0.84).

#### Outcome analysis—Daily analysis for the first 7 days from the time of injury

The total number of patients included in the outcome analysis was 182, as described in the previous paragraph.

**Fig. 7:**
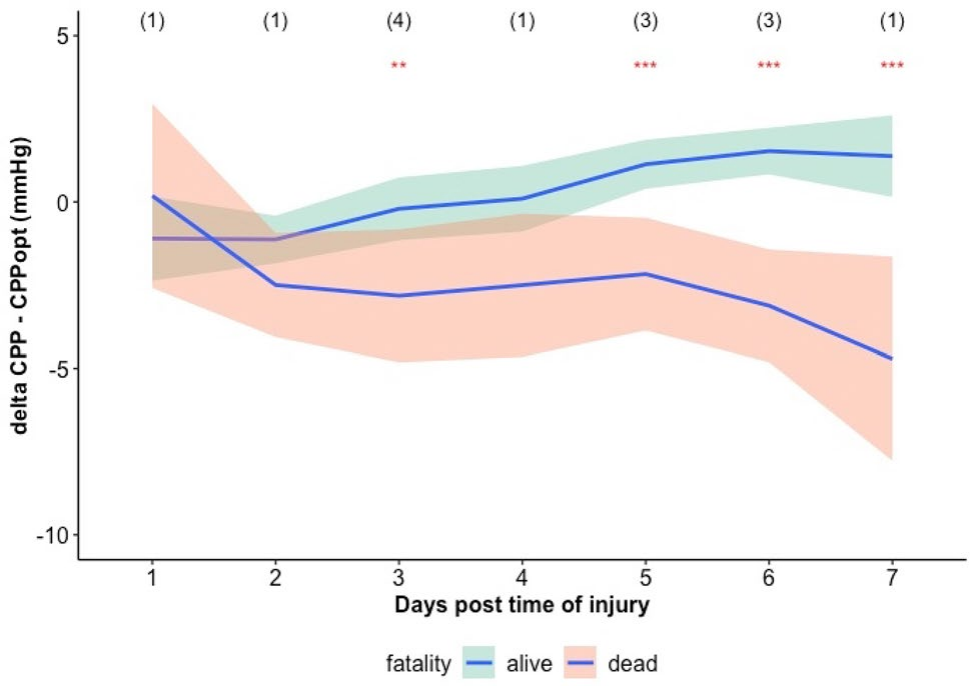
Trend of daily average DeltaCPPopt for mortality groups. The locally estimated scatterplot smoothing (LOESS) function was derived for DeltaCPPopt versus day post injury relationship for each mortality group. Outcome was assessed with GOSE at 6 months. Red asterisks indicate statistically significant difference in DeltaCPPopt between mortality groups (Kruskall-Wallis test, ***: p < 0.001 and **: p < 0.01). Numbers in brackets at the top indicate number of patients that died per each day. DeltaCPPopt: average deviation of cerebral perfusion pressure from optimal cerebral perfusion pressure assessed with the algorithm resulted after our fine-tuning process

Trends of daily average DeltaCPPopt for mortality groups are presented in Fig. 7. The difference between mortality groups is significant on the 3rd day and from day 5 to 7 (Kruskall-Wallis, *p* < 0.05).

Neither DeltaCPPopt (Kruskal–Wallis, *p* > 0.05) nor DeltaCPPopt_MA (Kruskal–Wallis, *p* > 0.05) could distinguish between favourable and unfavourable outcome on a daily basis.

## Discussion

In this study we describe the efforts that led to increase stability and reliability of the optimal cerebral perfusion pressure (CPPopt) trendline automated algorithm based on a multi-window approach. We confirmed that such adjusted CPPopt algorithm has increased stability and lower yield when compared to the previous one in a multi-centre cohort of traumatic brain injury (TBI) patients. We also validated the ability of CPPopt in discriminating mortality groups.

A new set of heuristics was chosen to ensure a greater stability and reliability of the automatically generated CPPopt during the fine-tuning process in the laboratory environment. The main technical differences from the previous algorithm and their rationale are discussed in Beqiri et al. 2021 [9]. Stability and reliability are important when the algorithm is used to assess CPPopt continuously at the bedside for individual patients. The algorithm presented in this work was adopted in the COGiTATE study [8]. COGiTATE clinical trial showed for the first time the safety and feasibility of targeting CPPopt prospectively in TBI patients admitted in ICU. In the intervention arm of the trial, CPP target recommendations were based on output of the CPPopt algorithm described in this manuscript, and were reviewed 4-hourly. CPPopt-based recommendations were provided in 74% of the 552 CPP reviews. ﻿The clinical team adopted this target in 92% of the reviews. The fact that COGiTATE could run successfully, and the final results of the study, indicate the achievements of the fine-tuning process.

The stability analysis corroborates the results of our previous single centre study [9] that included 840 TBI patients. The number of patients in the multicentre analysis presented in this manuscript is much smaller (less than 200 patients). However, its importance lies in the fact that the validation is performed in data coming from ICUs in 21 different hospitals in Europe [18]. Therefore, the management protocols of TBI patients would have higher heterogeneity as opposed to the management protocols coming from one centre. The time trends generated by the current algorithm proved to be more stable than the trends generated by the previous algorithm (see results, paragraph ‘stability and yield analysis’ and Table 2). Moreover, the difference in stability between the two methods is significant starting from the first 24 h of recording, and was not time-dependent (Fig. 4).

The restrictions added to the automated algorithm would likely decrease the availability of the CPPopt time trend in time (yield), which is an important issue for the clinical application of the concept. The yield of CPPopt was previously investigated in collaboration with Liberti et al. [24] in the same cohort of patients. The yield was 80.7% (IQR 70.9–87.4) for the whole CPP monitored period. These data suggested CPPopt was available during most of the monitored period during the ICU stay. In the same work, the authors showed that CPPopt availability was not related to demographic and injury severity admission variables, suggesting that CPPopt guided management could potentially be applied to any TBI patient requiring ICP directed therapy without restrictions depending on admission features. The yield of CPPopt was here reassessed for the first 7 days from the start of recording and compared to the yield of CPPopt_MA. Similarly to what Liberti et al. described, the yield of CPPopt was 80.5% (IQR 70- 87.5) and it was lower than the yield of CPPopt_MA as expected. We explored the relationship between yield and PRx and we observed that for high PRx values there was a linear relationship between the two variables. In, particular, the higher the PRx, the lower the yield. This might be related to the fact that for very high values of PRx maintained over a large span of time, the relationship between PRx and CPP would not show a U-shape curve. In fact, if autoregulation is uniformly impaired over the whole available CPP range, there is no indication that changing CPP will result in improvement of the autoregulation. Thus, there is no ‘optimum’ CPP for that period, leading to decreased ‘yield’. Similar effect may likely also occur for period with uniformly active CA, which might also be hinted by the slight downward slope of the yield plot for low values of PRx.

Finally, the relationship of the current CPPopt algorithm with outcome was previously studied in the same CENTER-TBI HR cohort in collaboration with Riemann et al. [10] The average deviation of CPP from CPPopt calculated for the whole monitored period was confirmed to be a predictor of mortality in both univariate and multivariate analysis including presentation and injury severity variables. In this study we showed that the current algorithm (CPPopt) performed no worse than the previous algorithm (CPPopt_MA) in predicting mortality, considering the first 7 days from the time of injury only. Here we didn’t investigate further outcome analysis, as the objective of this study was to establish that the heuristics introduced in the fine-tuning process did not have a detrimental effect in outcome prediction. The difference between mortality groups was significant starting from the third day (Fig. 7). The fact that CPPopt is available on average after 5 h from the beginning of the recording, highlights how CPPopt could be a useful digital biomarker available when it is most relevant. The implications of these findings are that CPPopt guided CPP management in TBI patients could potentially be implemented in ICUs of different countries on a large scale.

Despite the improvements in CPPopt methodology, a recent Delphi consensus of clinicians [25] concluded that ‘*consensus could not be reached on the accuracy, reliability and validation of any current CA assessment method. There was also no consensus on how to implement CA information in clinical management protocols, reflecting insufficient clinical evidence*’.

It is therefore deemed important at this point to step back and look at methods for improving the determination of autoregulatory metrics and the autoregulatory curve in individual TBI patients. The main question we must ask ourselves is: what are the reasons for the apparent underperformance of those metrics in terms of accuracy and reliability? These aspects require future investigation.

### Limitations

Our study has some limitations. First, the statistical validation of this study is a retrospective investigation. Second, given the small number of patients in a multi-centre study, there was an uneven distribution of numbers of recruited patients per centre. Third, patients with decompressive craniectomy and patients with ICP monitored via EVD were excluded in our validation study, as careful considerations are required for the calculations of PRx here. Whether or not the presented methodology is applicable in those condition is still a matter of debate and requires further investigation. Fourth, PRx describes a global cerebrovascular reactivity, given that it relies on how ICP reflects global changes in blood volume. Hence, differences between patients with unilateral or focal injury versus bilateral or diffuse injury are not captured in our analysis.

## Conclusion

CPPopt automated algorithm based on a multiwindow approach was implemented with a new set of heuristics chosen to ensure a greater stability and reliability of the output values during the fine-tuning process in the laboratory environment. The current, tuned, algorithm confirms its higher stability when compared to the previous one in a multi-centre cohort of TBI patients. The yield was indeed reduced, but remained close to 80%. Finally, CPPopt maintained the ability of discriminating outcome groups not worse than the previous algorithm.
